# Functional Characterization and Toxicological Study of *Cordyceps militaris* in Weaned Pigs

**DOI:** 10.3390/toxins16120507

**Published:** 2024-11-25

**Authors:** Yanping Li, Yang Lu, Bing Yu, Zhiqing Huang, Yuheng Luo, Ping Zheng, Xiangbing Mao, Jie Yu, Junqiu Luo, Hui Yan, Jun He

**Affiliations:** 1 Institute of Animal Nutrition, Sichuan Agricultural University, Chengdu 611130, China; yanpingli@163.com (Y.L.); ybingtian@163.com (B.Y.); z.q.huang@163.com (Z.H.); luoluo212@126.com (Y.L.); zpind05@163.com (P.Z.); acatmxb2003@163.com (X.M.); jerryyujie@163.com (J.Y.); 13910@sicau.edu.cn (J.L.); yan.hui@sicau.edu.cn (H.Y.); 2 Key Laboratory of Animal Disease-Resistant Nutrition, Chengdu 611130, China; 3 Institute of Animal Husbandry & Veterinary Sciences, Shanghai Academy of Agricultural Sciences, Shanghai 201106, China

**Keywords:** *Cordyceps militaris*, weaned pigs, safety, growth performance

## Abstract

*Cordyceps militaris* (CM), a well-known parasitic fungus that grows on the larvae of *Lepidoptera*, has a variety of pharmacological activities. However, little is known about its safe dosage for animals, including pigs. To explore its effect on intestinal health and evaluate its safe dosage, 30 weaned pigs were randomly allotted to five groups and fed with a basal diet supplemented with different doses of CM for 42 days. The results showed that CM supplementation at 100 mg/kg increased the average daily weight gain (ADG) and significantly decreased the ratio of feed intake to gain (F:G) in the weaned pigs (*p* < 0.05). However, CM supplementation at a higher dose (1000 to 4000 mg/kg) had no effect on growth performance. CM supplementation at 100 mg/kg also increased the digestibility of gross energy and increased the ratio of villus height to crypt depth (V/C) in the duodenum and ileum (*p* < 0.05). Moreover, CM supplementation at 100 mg/kg increased the activities of catalase (CAT) and total antioxidant capacity (T-AOC), but decreased the concentration of malondialdehyde (MDA) in serum (*p* < 0.05). Importantly, histopathological studies of tissues (e.g., heart, liver, kidney, spleen, lungs, pancreas, thymus, mesenteric lymph nodes, stomach, and small intestine), organ indexes, major hematological parameters, and serum biochemical parameters were not affected upon CM supplementation. These results suggest that CM may have the potential to act as a safe and effective supplement to improve the growth performance and intestinal health of weaned pigs.

## 1. Introduction

*Cordyceps militaris* (CM), an entomopathogenic fungus of the genus *Cordyceps* in the *Cordycepsaceae* family [1], can act as a health product in East Asia due to its nutritional and medicinal value [2]. A great variety of active ingredients with pharmacological activity have been isolated from CM, including cordycepin, cordyceps polysaccharide, ergosterol (a sterol present in fungi), carotenoids, D-mannitol, essential amino acids, and volatile oils [3]. Currently, CM has been implicated in regulating a wide variety of biological functions, including hypolipidemic, antitumor, anti-microbial, antioxidant, anti-metastatic, anti-viral, anti-inflammatory, neuroprotective, and immuno-protective activities [2,4]. For instance, CM exhibited strong antibacterial and antifungal properties in an in vitro study [5]. Previous studies have also indicated that spent mushroom (*Cordyceps militaris*) can improve the growth performance and health status of Nile tilapia [6]. Dietary CM supplementation at 300 mg/kg can enhance intestinal barrier function and repress inflammation in growing pigs [7]. Moreover, CM has been reported to regulate the immune system in humans [8] and alleviate DSS-induced ulcerative colitis in mice by regulating the intestinal mucosal barrier and gut microbiota [9]. Although a number of studies have been performed, there is no clear-cut evidence showing the exact relationship between the dose and effects of CM, as the effects of CM on animals are closely related to animal species, dosage, and the physiological stage of animals.

In experimental rats, the administration of CM at 3600 mg/kg/day for 90 days did not cause any toxicologically significant treatment-related changes in clinical, histopathological, or hematological parameters [10]. In human studies, CM supplementation did not correlate with the occurrence of serious adverse events [11]. However, the known compounds isolated from *cordyceps* fungi include the adenosine analogs cordycepin and pentostatin, which have been reported to have different beneficial or pharmaceutical activities and dose-dependent cytotoxicities, neurological toxicities, and toxicological effects in both human and animals [12]. Cordycepin (3′-deoxyadenosine, C10H13N5O3), a water-insoluble organic compound, is a nucleoside derivative purified from CM [13]. Previous studies have shown that the acute toxicity symptoms resulting from cordycepin mainly include nausea and diarrhea [12]. *Cordyceps militaris* polysaccharides are the major bioactive substances of CM, comprising three main monosaccharides, mannose, glucose, and galactose with a backbone characterized by (1→4)-β-D-Glcp, (1→6)-linkedα-D-Gl*up*, (1→3)-β-D-Gl*cp*,and (1→2)-α-D-Ma*np* [14,15]. Currently, numerous studies have confirmed the beneficial effects of *Cordyceps militaris* polysaccharides on human health. For example, studies have shown that *Cordyceps militaris* polysaccharides have free radical scavenging activities [5]. But our literature review found that no studies have analyzed the toxicity and safety of polysaccharides from CM. Therefore, it is of great importance to evaluate the safety of CM for both human and animals. However, little is known about the safe dosage of CM for young animals like piglets. The aim of this study was to explore the safety of dietary CM supplementation from an animal toxicology perspective. Our findings may also be important for those who like to consume CM.

## 2. Results

### 2.1. Growth Performance and Diarrhea Rate

There were no unexpected deaths during the study. The effects of dietary CM supplementation on the growth performance and diarrhea rate of pigs are presented in Table 1. There were no differences in the final body weight (FBW), average daily feed intake (ADFI), and diarrhea rate between the CM groups and the CON group (*p* > 0.05). However, supplementing 100 mg/kg CM in the diet significantly increased the ADG, but decreased the F/G ratio (*p* < 0.05). Compared with the CON group, dietary CM supplementation had no influence on the ADG and F/G ratio of the weaned pigs. In addition, the final ADG showed linear and quadratic responses to the increasing dietary CM levels (linear, *p* < 0.05; quadratic, *p* < 0.05).

### 2.2. Nutrient Digestibility

The results of the apparent total tract digestibility of nutrients are shown in Table 2. Dietary 100 mg/kg CM supplementation significantly increased the digestibility of gross energy (GE) (*p* < 0.05). As compared to the control group, high dietary doses of CM (1000–4000 mg/kg) supplementation had no effect on the nutrient digestibility of GE and dry matter (DM) (*p >* 0.05). Moreover, there were no differences in the digestibilities of ash, crude protein (CP), or ether extract (EE) between the control group and the CM-supplemented groups (*p* > 0.05). In addition, the nutrient digestibility of DM and GE showed linear and quadratic responses to the increasing dietary CM levels (linear, *p* < 0.05; quadratic, *p* < 0.05).

### 2.3. Intestinal Morphology

The intestinal morphology of the pigs is shown in Table 3. Dietary CM supplementation had no influence on villus height or crypt depth in the small intestine (*p* > 0.05). However, dietary supplementation with 100 mg/kg CM significantly increased the ratio of V/C in the duodenum and ileum (*p <* 0.05).

### 2.4. Blood Routine Indices

The routine blood indices of the piglets are presented in Table 4. There were no differences in hematological parameters at the beginning of the experiment among the five treatments (*p* > 0.05). On d 43, dietary 100 mg/kg CM supplementation increased the number of eosinophils (EOS) (0.05 < *p* < 0.10).

### 2.5. Serum Biochemical Indices

The serum biochemical parameters of the piglets are presented in Table 5. There were no significant differences in the serum biochemical indices at the beginning of the trial (*p* > 0.05). On day 43, supplementation with 100 mg/kg CM in the diet tended to decrease the serum ALP activities (0.05 < *p* < 0.10). However, dietary CM supplementation had no influence on the contents of UREA or GLU and other serum biochemical parameters.

### 2.6. Serum Antioxidant Indices

The serum antioxidant indices of the piglets are represented in Table 6. There were no significant differences in the serum antioxidative parameters at the beginning of the trial. On day 43, dietary 100 mg/kg CM supplementation significantly increased the serum concentrations of CAT and T-AOC, but decreased the serum concentration of MDA in the weaned pigs (*p <* 0.05). As compared to the control group, high dietary doses of CM (1000–4000 mg/kg) supplementation had no influence on the serum antioxidative parameters (*p >* 0.05).

### 2.7. Relative Organ Weights

The results for the relative weights of the pigs’ organs are presented in Table 7. Dietary CM supplementation had no effect on the relative organ weights, including the liver, kidney, spleen, heart, pancreas, and lungs (*p* > 0.05).

### 2.8. Organ Pathobiology

**Figure 1:**
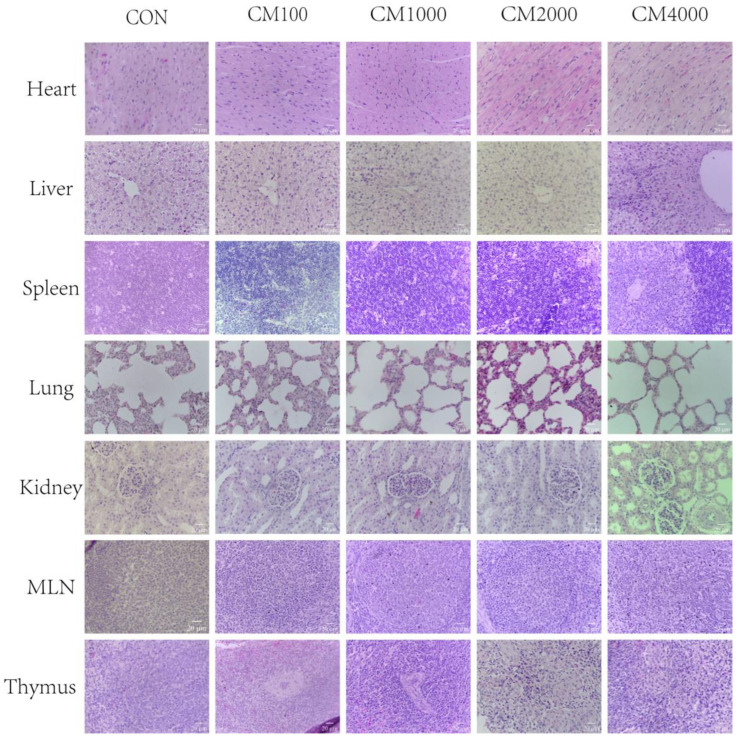
Effects of dietary CM supplementation on organ histopathology of heart, liver, kidney, spleen, thymus, mesenteric lymph nodes, and lungs in piglets (H&E; ×400). CON, basal diet fed; CM100, CM1000, CM2000, and CM4000, pigs were fed a basal diet supplemented with 100, 1000, 2000, or 4000 mg/kg CM; and MLN, mesenteric lymph nodes.

**Figure 2:**
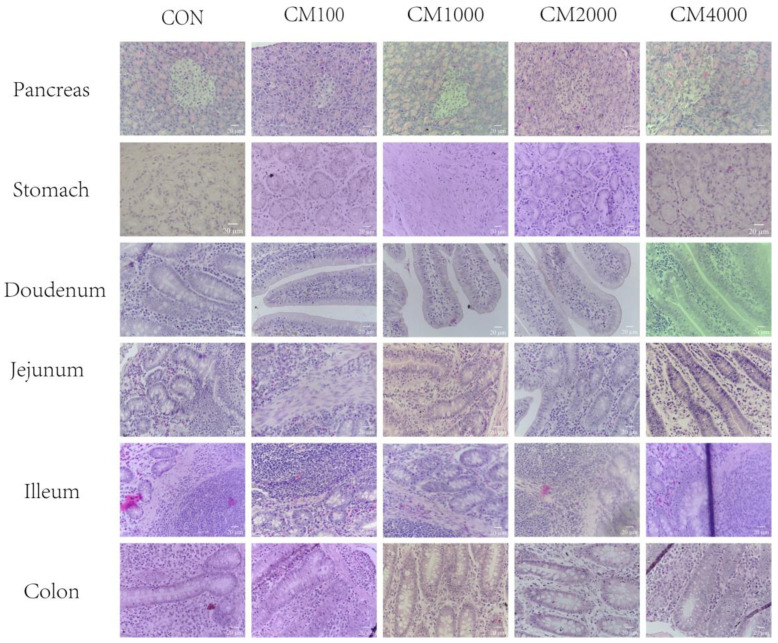
Effects of dietary CM supplementation on organ histopathology of pancreas, stomach, duodenum, ileum, jejunum, and colon in piglets (H&E; ×400). CON, basal diet fed; CM100, CM1000, CM2000, and CM4000, pigs were fed a basal diet supplemented with 100, 1000, 2000, or 4000 mg/kg CM.

The influences of CM supplementation on organ pathobiology are presented in Figure 1 and Figure 2. The results showed that dietary CM supplementation had no effects on the morphology of any of the organs, including the heart, liver, kidney, spleen, lungs, pancreas, thymus, mesenteric lymph nodes, stomach, duodenum, jejunum, ileum, and colon (Figure 1 and Figure 2). The structure of the cardiac muscle fibers and the cardiomyocytes were normal, and there was no inflammatory cell infiltration; the hepatocytes were normal, the liver lobule structure and hepatic cord were clear, and there was no inflammatory cell infiltration; the red pulp region of the spleen had no obvious congestion, and the number and size of lymphoid follicles in the white pulp area were normal; the kidney tubules and glomerulus were normal; the structure of the mucosal layer and the arrangement of the intestinal villi were normal; the structure and size of the alveoli were normal, with no obvious lesions seen; the morphologies of pancreatic islets A, B cells, and adipocytes were normal; and no other obvious lesions were found.

**Figure 3:**
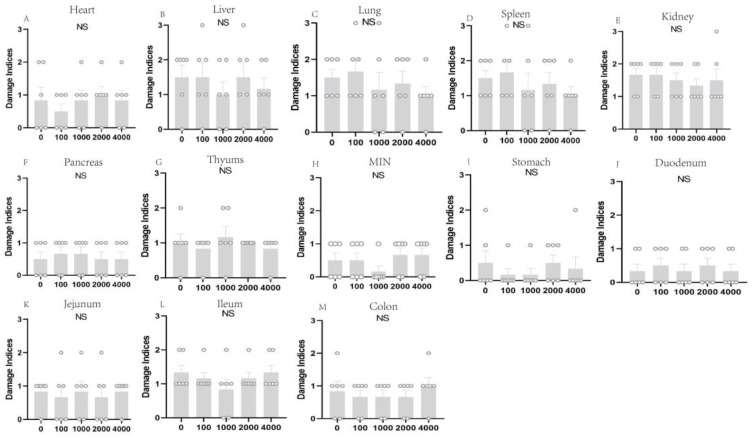
Effect of different CM supplementation levels on organ/tissue damage indices in pigs. (**A**–**M**) Damage degree of heart, liver, kidney, spleen, thymus, mesenteric lymph nodes, lungs, pancreas, stomach, duodenum, ileum, jejunum, and colon in pigs. The damage indices range from 0 to 3 (0, completely healthy; 1, mild injury; 2, moderate injury; and 3, severe injury). Each dot represents the organ damage score of an individual pig (*n* = 6). NS, not significant (*p* > 0.05).

As shown in Figure 3, there were no significant differences in the organ histopathology analyses among the five treatments according to the histology score (organ damage index) (*p* > 0.05).

## 3. Discussion

*Cordyceps militaris* is a unique medicinal tonic fungus with a variety of pharmacological activities [3]. CM and its byproducts have recently been utilized as feed additives to enhance the immunomodulatory activity, growth performance, and antioxidant capacity in animals [16,17]. For example, a study on broiler chickens reported that CM could increase body weight gain [17]. However, the dose effect and the potential detriments of CM used as feed additives are unknown. In this study, the highest dosage of CM added to the feed of piglets was 4000 mg/kg, which is 40 times its effective dose (100 mg/kg). The results of our research may contribute to the future development of CM and its associated by-products for use in food, traditional medicine, or animal feed. In our study, we found that diets supplemented with 100 mg/kg CM improved growth performance, as shown by a decreased F:G ratio and increased ADG, which is in accordance with previous studies showing that diets supplemented with 1 g/kg fermented *Cordyceps militaris* improved the growth performance of broiler chickens [16]. However, we also found that dietary CM supplementation at a higher dose (1000–4000) had no effect on the growth performance of piglets, which is in accordance with previous studies on Nile tilapia showing that excessive dietary CM supplementation resulted in a decrease in the fish innate immune response and subsequently a poor growth performance [6].

The intestine plays a pivotal role in the digestion and absorption of nutrients and is composed of villi and crypts covered by a single layer of columnar epithelial cells [18]. Therefore, the intestinal morphological structure is highly related to the nutrient digestion, absorption capacity, and growth performance of animals [19]. Previous studies have shown that CM exerted beneficial effects on the intestinal morphology of pigs and mice [7,20]. A study on dextran sodium sulfate (DSS)-injured mice showed that supplementation with CM could enhance the integrity of the intestinal mucosal barrier by up-regulating MUC2 and TJ proteins [9]. In this study, we also found that diets supplemented with 100 mg/kg CM not only exerted a positive effect on intestinal morphology, but also increased the digestibilities of ASH and GE. The elevated growth performance mentioned above may be attributed to improvements in the absorption and digestion of nutrients. However, we also found that dietary CM supplementation at a higher dose (1000–4000) did not affect intestinal morphology. Previous studies observed that an excess intake of prebiotics had a detrimental influence on intestinal enterocytes [6]. Consistent with the side effects of adenosine analogs [21,22], some studies have indicated that excessive cordycepin intake might cause nausea and diarrhea, which might also partly explain this phenomenon.

Evaluations of hematological parameters are important for showing the common clinical manifestations of toxic reactions within the body [23]. In this study, CM supplementation had no effect on the major routine blood parameters of the piglets and all routine blood parameters were within the normal physiological range for weaned pigs, which is in accordance with previous studies on mice and rodents [10,11]. The results indicated that CM supplementation was safe and did not adversely affect the hematological indicators of the pigs. Eosinophils are responsible for the immune response in tissues, which can quickly consume pathogens that invade the host [24,25]. In this study, diets supplemented with 100 mg/kg CM significantly increased the concentrations of eosinophils. These results suggest that CM supplementation had an anti-inflammatory effect on the weaned pigs. However, we also found that dietary CM supplementation at a higher dose (1000–4000) did not affect the concentrations of eosinophils. The possible explanations for the effects of cordycepin on the mTOR pathway might be that high doses of cordycepin suppress the immune system, as does the mTOR inhibitor rapamycin (sirolimus) [26].

Serum biochemical parameters can well reflect an animal’s health condition and metabolic status [18]. AST and ALT are intracellular enzymes that are regarded as biomarkers of acute liver damage [27]. Moreover, the concentrations of TBIL, ALB, and TP are also used as important markers for liver diseases [28]. In this study, there were no significant differences in these serum biochemical parameters, which is consistent with a previous study on rats [10]. These results indicate that diets supplemented with different levels of CM had no adverse effect on liver function in weaned piglets. ALP is an intracellular enzyme existing in the apical microvilli and is considered as an essential intestinal mucosal defense factor for maintaining intestinal homeostasis [29]. In this study, diets supplemented with 100 mg/kg CM tended to decrease the concentration of ALP, indicating that CM supplementation is beneficial for improving intestinal barrier integrity. Serum creatinine and urea are commonly used renal markers that are released into the circulation system upon kidney damage [30,31]. In this study, dietary CM supplementation did not affect the serum creatinine and urea levels. This result suggests that diets supplemented with different levels of CM had no adverse effect on the normal healthy structure of the renal system.

Oxidative stress, a consequence of imbalances in oxidative and antioxidative systems, can lead to tissue damage [32]. The antioxidant capacity of CM was observed in mice and chickens [5,17]. The presence of antioxidant molecules from CM, such as δ-tocopherol, *p*-hydroxybenzoic acid, and *Cordyceps militaris* polysaccharides, may be related to its antioxidant activity [5]. For instance, previous studies found that polysaccharides extracted from CM exhibited strong hydroxyl, superoxide, and 1-diphenyl-2-picrylhyrazyl (DPPH) radical scavenging activity [33,34]. T-AOC, an oxidative stress and antioxidant defense biomarker, reflects the antioxidant capacity [35]. CAT is a critical antioxidative enzyme in the body responsible for eliminating ROS [36]. MDA, a lipid peroxidation product, is widely considered as a biomarker of oxidative stress [37]. In our study, pigs fed with 100 mg/kg CM had greater CAT and T-AOC activities and a lower MDA content in their serum, which is in accordance with our previous study [38]. Previous studies have shown that oxidative stress disrupts tight junctions and activates apoptosis in intestinal epithelial cells, thereby undermining the integrity of the intestinal barrier [39]. This indicates that a proper dosage of CM can increase the antioxidant capacity of the body, which may partly explain the improvements in intestinal functions mentioned above.

The relative weight of an organ is a crucial parameter in toxicological studies [40,41]. In this study, the relative weights of organs, including the heart, lungs, spleen, liver, pancreas, and kidney, did not differ significantly among the five treatments. To further explore the effects of dietary CM on pig organs, we determined the histopathology for a total of 12 organs. In this study, there was no evidence of gross abnormalities in the histopathological examinations between the treatment and control groups. Moreover, we employed histopathological scoring to determine the effects of dietary CM on the pig organs and tissues. Our results found that CM did not influence the histopathological scoring of organs, even at a 4000 mg/kg supplementation level with a long experimental period. These results were consistent with the hematological parameters and serum biochemical parameters, indicating that CM added to the diet of young pigs at a dose lower than 4000 mg/kg is safe and has no major adverse effect on piglets.

## 4. Conclusions

In conclusion, CM has no toxic effects at a dose below 4000 mg/kg, as indicated by the pigs’ normal serum clinical chemistry, hematological parameters, and organ histopathology. Meanwhile, diets supplemented with 100 mg/kg CM improved growth performance and nutrient utilization, which was associated with an improved intestinal health and antioxidant capacity. Thus, diets supplemented with 100 mg/kg CM may be a feasible option for weaned pigs with favorable effects.

## 5. Materials and Methods

All experimental protocols used in the animal experiment were approved by the Institutional Animal Care and Use Committee of Sichuan Agricultural University (Authorization number SICAU-2022-014).

### 5.1. Experimental Design, Animals, and Diet

A total of 30 male Duroc × Landrace × Yorkshire pigs weaned at 24 d (with an average initial body weight (IBW) of 7.22 ± 0.41 kg) were used for the trial. The pigs were randomly allotted into five dietary treatments according to their initial BW. The experiment lasted for 42 days and the pigs in different groups were exposed a basal diet supplemented with 100, 1000, 2000, and 4000 mg/kg CM, respectively. These CM levels were acquired by additions of 100, 1000, 2000, or 4000 mg/kg CM to the basal diet. CM, with the main ingredients of cordycepin (≥1%) and polysaccharides (≥30%), was obtained from Shanghai Academy of Agricultural Sciences (Shanghai, China). The basal diet (Table 8) was formulated according to the National Research Council [42] recommendations to meet and slightly exceed the swine nutrient requirements. All pigs were individually housed in a 1.5 × 0.7 m^2^ metabolism cage with the room temperature controlled between 25 and 28 °C, relative humidity at 65% ± 5%, and had free access to feed and fresh water throughout this 42 d experimental period.

### 5.2. Dose Selection and Homogenization of CM

The concentrations of CM in the diets were 0, 100, 1000, 2000, and 4000 mg/kg, which was decided according to our previous study on piglets and the request of the Ministry of Agriculture and Rural Affairs. There were two steps involved in homogenizing the CM. Firstly, the CM was accurately weighed and diluted with the carrier (extruded corn). Secondly, the primary premix was added to other raw ingredient powders.

### 5.3. Sample Collection

Feed samples from each treatment were individually collected in sample bags after the feeds were prepared and sorted at −20 °C. During days 39–42 of the trial, fresh fecal matter from each pig was immediately sampled in each cage and added to a 10% H_2_SO_4_ solution (10 mL of solution per 100 g of the fresh fecal matter) with a few drops of toluene. The feed and fecal samples were dried at 65 °C for 72 h, milled into powder to pass through 40-mesh sieves, and sorted at −20 °C for nutrient analysis. At the beginning (day 1) and the end (day 43) of the experiment, blood samples were obtained from each pig by jugular vein puncture after 12 h of fasting. In total, 5 mL of blood was collected into heparinized tubes with a minimum amount of stress for routine blood indices analysis, and another 15 mL of blood was injected into vacuum tubes and centrifuged at 3500× *g* for 15 min at 4 °C to obtain serum, which was stored at −20 °C, for serum indices analysis. On day 43 of the experiment, all pigs were slaughtered after electrical stunning to obtain samples of their organs and entire intestines. The middle sections (4 cm) of the duodenum, jejunum, ileum, and cecum were gently rinsed with cold phosphate-buffered saline (PBS) and fixed in 4%paraformaldehyde solution for morphological analysis. In addition, tissue samples of about 5 cm^3^ from each organ were collected at the same sites and kept in the same way for morphological assessment (heart, liver, kidney, spleen, lungs, pancreas, thymus, lymph nodes, and stomach).

### 5.4. Growth Performance and Diarrhea Rate Evaluation

On days 1 and 43 of the experiment, all pigs were weighed after 12 h of fasting to obtain their initial body weight (IBW) and final body weight (FBW), and the feed intake of each pig was recorded during the trial. These data were used to calculate the average daily feed intake (ADFI), average daily gain (ADG), and feed efficiency (G:F) of the pigs.

The diarrhea state was observed and recorded twice daily each day during the experimental period. Fecal consistency was scored as follows: 0, normal; 1, soft feces; 2, semiliquid feces; and 3, watery feces. The piglets were identified as having diarrhea when their fecal consistency score was ≥2. The diarrhea rate was calculated in accordance with previous methods [43], as follows: diarrhea rate (%) = (the number of pigs with diarrhea days × of diarrhea)/(total number of piglets × the number of experimental days) × 100.

### 5.5. Apparent Total Tract Nutrient Digestibility Analysis

The milled diet and fecal samples were prepared for nutrient digestibility analysis, which used Cr_2_O_3_ as an external indicator. The dry matter (DM), ash, crude protein (CP), and ether extract (EE) contents were analyzed following the procedures of the Association of Official Agricultural Chemists (AOAC) [44]. The gross energy (GE) content was detected using an adiabatic bomb calorimeter (LECO, St. Joseph, Michigan, USA). The apparent total tract digestibility was calculated using the following equation: ATTD (%) = 100 − 100 × (digesta nutrient × diet Cr_2_O_3_)/(diet nutrient × digesta Cr_2_O_3_) [45].

### 5.6. Routine Blood and Biochemical Indices

Routine blood indices, including the white blood cell count (WBC), neutrophils count (Neut), lymphocytes count (Lymph), monocytes count (Mono), eosinophils count (Eos), basophils count (Bas), red blood cell count (RBC), hemoglobin (HGB), hematocrit (HCT), red blood cells, mean corpuscular volume (MCV), red cell distribution width-SD (RDW-SD), red cell distribution width-CV (RDW-CV), mean hemoglobin (MCH), mean corpuscular hemoglobin concentration (MCHC), platelet (PLT), and thrombocytosis (PCT), were determined using an automated hematology analyzer (KX-21, SYSMEX, Wuxi, China).

Ten blood biochemical indices, including total protein (TP), glucose (GLU), albumin (ALB), alkaline phosphatase (ALP), creatinine (CREA), alanine transaminase (ALT), aspartate transaminase (AST), bilirubin total (TBIL), globulin (GLO), and blood urea nitrogen (BUN), were measured by an automatic biochemistry analyzer (7150, HITACHI, Tokyo, Japan).

The antioxidant indices in the serum, including glutathione peroxidase (GSH-PX), superoxide dismutase (SOD), malondialdehyde (MDA), catalase (CAT), and total antioxygenic capability (T-AOC), were determined by specific commercial assay kits (Nanjing Jiancheng Bioengineering Institute, Nanjing, China). All procedures were strictly performed according to the manufacturer′s instructions.

### 5.7. Relative Organ Weights

Following slaughtering, the piglets were properly bled. All the organs (including the liver, kidney, spleen, heart, pancreas, and lungs) were weighed immediately to obtain their relative weights. All superficial fat or blood on the tissues was removed before weighing. The relative organ weight was calculated by the following equation: A1/A2 [46]. A1: organ weight (g); A2: preslaughter weight of piglet (kg).

### 5.8. Intestinal Morphology and Organ Pathobiology

The collected organs and tissue samples were fixed in a 4% paraformaldehyde solution for 24 h. The fixed organs and tissue samples were dehydrated through a graded series of ethanol, embedded in paraffin wax, sliced into tissue sections, stained with hematoxylin and eosin (H&E), and sealed with resin for further assessment. Samples were observed at a 100× to 400× magnification to determine the morphological structures, and a minimum of 10 visions were observed over each sample. All sections were examined and graded by a well-experienced veterinary pathologist. For the heart, liver, kidneys, spleen, lungs, pancreas, thymus, mesenteric lymph nodes, stomach, and intestines, qualitative scorings with respective scores and grading standards were used to evaluate the health status of the pigs. The score of the section was the highest score of the pathological changes observed in each section. For example, if pathological change scores of 1 and 2 were observed at the same time in a field, the score of this section was 2. The average values of the scores of all the sections in one group would be the viscera damage indices of the treatment, ranging from 0 to 3.

The method of measurement for crypt depth and villus height was referred to previous methods [47]. A minimum of 10 intact villi and crypts for each intestinal sample were analyzed using a microscope (BX41, Olympus Corporation, Tokyo, Japan) and an analysis system (Media Cybernetics, Bethesda, MD, USA). Then, the villus height-to-crypt depth ratio (VCR) was calculated.

### 5.9. Statistical Analysis

All data were analyzed by one-way analysis of variance (ANOVA) with SPSS 24.0 (IBM, Chicago, IL, USA), and differences among treatments were detected using Duncan’s method. *p* < 0.05 was considered to be a significant difference, and 0.05 < *p* < 0.1 was considered to be a tendency. The results are expressed as means with standard errors.

## Figures and Tables

**Table 1:** Effects of CM on growth performance and diarrhea rate in weaned pigs.

Items		CM (mg/kg)				SEM	*p* Value		
	CON	100	1000	2000	4000		ANOVA	Linear	Quadratic
IBW (kg)	7.27 ± 0.47	7.20 ± 0.48	7.27 ± 0.41	7.22 ± 0.35	7.15 ± 0.43	0.07	0.988	0.698	0.854
FBW (kg)	24.2 ± 1.62	25.4 ± 1.81	25.0 ± 1.63	23.2 ± 2.56	23.1 ± 2.76	0.40	0.263	0.125	0.399
ADFI g/d	793 ± 56.6	791 ± 99.5	821 ± 66.8	759 ± 55.4	749 ± 130.9	15.49	0.624	0.291	0.429
ADG g/d	394 ± 21.9 ^b^	438 ± 37.4 ^a^	410 ± 23.8 ^ab^	356 ± 14.1 ^b^	379 ± 58.2 ^ab^	8.50	0.019	0.040	0.034
F/G	2.02 ± 0.07 ^a^	1.80 ± 0.10 ^b^	1.98 ± 0.15 ^ab^	2.09 ± 0.17 ^a^	2.02 ± 0.07 ^a^	0.03	0.012	0.105	0.012
diarrhea rate %	11.7 ± 2.05	10.5 ± 3.23	9.5 ± 2.50	13.1 ± 3.61	12.7 ± 1.62	5.21	0.592	0.430	0.532

IBW, initial body weight; FBW, final body weight; ADFI, average daily feed intake; ADG, average daily gain; F/G, feed to gain ratio; and CON, basal diet fed. Data are listed as means with SEM (*n* = 6). ^a,b^ mean values with unlike superscript letters were significantly different(*p* < 0.05).

**Table 2:** Effects of CM on ATTD of nutrients in weaned pigs.

Items		CM (mg/kg)				SEM	*p* Value		
	CON	100	1000	2000	4000		ANOVA	Linear	Quadratic
DM, %	0.83 ± 0.10 ^ab^	0.84 ± 0.04 ^a^	0.84 ± 0.09 ^ab^	0.82 ± 0.09 ^b^	0.82 ± 0.02 ^b^	0.03	0.018	0.011	0.052
GE, %	0.83 ± 0.01 ^b^	0.85 ± 0.03 ^a^	0.84 ± 0.08 ^ab^	0.83 ± 0.10 ^b^	0.83 ± 0.02 ^b^	0.03	0.024	0.037	0.049
Ash, %	0.40 ± 0.03	0.43 ± 0.02	0.39 ± 0.04	0.38 ± 0.03	0.40 ± 0.03	0.01	0.177	0.073	0.753
CP, %	0.78 ± 0.02	0.80 ± 0.02	0.79 ± 0.01	0.78 ± 0.02	0.78 ± 0.02	0.04	0.125	0.102	0.199
EE, %	0.71 ± 0.04	0.75 ± 0.04	0.73 ± 0.02	0.70 ± 0.03	0.72 ± 0.03	0.07	0.173	0.363	0.289

DM, dry matter; CP, crude protein; EE, ether extract; GE, gross energy; ATTD, apparent total tract digestibility; and CON, basal diet fed. Data are listed as means with SEM (*n* = 6). ^a,b^ mean values with unlike superscript letters were significantly different (*p* < 0.05).

**Table 3:** Effect of CM on intestinal morphology in the weaned pigs.

Items		CM (mg/kg)				SEM	*p* Value		
	CON	100	1000	2000	4000		ANOVA	Linear	Quadratic
Duodenum									
Villus height, μm	200 ± 27.9	202 ± 24.8	188 ± 32.1	211 ± 24.1	214 ± 9.89	4.55	0.417	0.257	0.335
Crypt depth, μm	127 ± 22.5	107 ± 14.7	112 ± 14.1	120 ± 16.0	125 ± 9.4	3.06	0.159	0.652	0.036
V/C	1.58 ± 0.12 ^c^	1.90 ± 0.14 ^a^	1.67 ± 0.14 ^bc^	1.77 ± 0.14 ^ab^	1.71 ± 0.09 ^bc^	0.03	0.003	0.437	0.035
Jejunum									
Villus height, μm	171 ± 30.8	175 ± 32.3	175 ± 20.5	173 ± 15.9	176 ± 22.2	4.36	0.998	0.792	0.945
Crypt depth, μm	93.9 ± 7.23	85.6 ± 10.01	88.0 ± 10.1	87.5 ± 6.1	83.0 ± 11.6	1.73	0.371	0.107	0.754
V/C	1.82 ± 0.24	2.03 ± 0.19	2.00 ± 0.25	1.98 ± 0.07	2.13 ± 0.12	0.04	0.128	0.031	0.699
Ileum									
Villus height, μm	186 ± 28.0	215 ± 26.3	205 ± 15.1	208 ± 28.5	185 ± 25.6	8.74	0.153	0.769	0.023
Crypt depth, μm	104 ± 14.7	99.2 ± 9.67	109 ± 10.1	112 ± 10.5	100 ± 12.3	2.15	0.319	0.710	0.299
V/C	1.79 ± 0.08 ^b^	2.16 ± 0.10 ^a^	1.89 ± 0.26 ^b^	1.86 ± 0.15 ^b^	1.84 ± 0.15 ^b^	0.04	0.005	0.366	0.033

V/C, villus height to crypt depth; CON, basal diet fed. Data are listed as means with SEM (*n* = 6). ^a,b,c^ mean values with unlike superscript letters were significantly different (*p* < 0.05).

**Table 4:** Effects of CM on blood routine examination in weaned pigs.

Items		CM (mg/kg)				SEM	*p* Value		
	CON	100	1000	2000	4000		ANOVA	Linear	Quadratic
Day1									
WBC (10^9^/L)	23.6 ± 9.98	27.2 ± 5.94	20.4 ± 4.83	18.7 ± 5.25	22.7 ± 7.81	1.31	0.302	0.263	0.589
Neut (10^9^/L)	5.85 ± 3.42	10.1 ± 3.32	8.46 ± 3.04	6.01 ± 3.23	8.80 ± 2.44	1.00	0.108	0.654	0.441
Lymph (10^9^/L)	1.19 ± 0.64	1.77 ± 0.38	1.39 ± 0.78	1.50 ± 1.22	2.26 ± 0.88	0.18	0.311	0.101	0.499
Mono (10^9^/L)	8.54 ± 4.32	10.3 ± 5.87	8.34 ± 5.07	5.71 ± 3.18	11.0 ± 5.73	4.95	0.418	0.939	0.373
Eos (10^9^/L)	0.43 ± 0.18	0.45 ± 0.11	0.22 ± 0.11	0.30 ± 0.16	0.40 ± 0.20	0.03	0.456	0.386	0.220
Bas (10^9^/L)	0.29 ± 0.19	0.25 ± 0.05	0.23 ± 0.07	0.32 ± 0.29	0.26 ± 0.12	0.05	0.871	0.982	0.810
RBC (10^9^/L)	7.01 ± 0.50	6.54 ± 0.39	6.64 ± 0.37	6.80 ± 0.68	6.45 ± 0.24	0.86	0.271	0.160	0.673
HGB (g/L)	115 ± 5.34	109 ± 8.87	109 ± 6.60	111 ± 8.48	104 ± 5.46	2.22	0.136	0.044	0.820
HCT (%)	39.2 ± 1.82	38.3 ± 3.63	37.6 ± 2.18	38.1 ± 2.20	35.8 ± 2.34	0.47	0.237	0.041	0.700
MCV (fL)	56.1 ± 2.57	58.5 ± 3.70	56.7 ± 1.54	56.4 ± 5.54	55.5 ± 2.67	0.61	0.665	0.483	0.362
RDW-SD (fL)	39.8 ± 1.77	40.0 ± 2.55	41.3 ± 2.54	43.3 ± 4.47	40.2 ± 1.32	0.57	0.215	0.252	0.159
RDW-CV (%)	20.0 ± 0.60	19.4 ± 0.81	20.6 ± 1.00	21.3 ± 3.00	20.5 ± 1.03	0.39	0.251	0.121	0.663
MCH (pg)	16.6 ± 0.67	16.7 ± 0.59	16.4 ± 0.41	16.5 ± 1.75	16.3 ± 0.59	0.16	0.955	0.541	0.756
MCHC (g/L)	295 ± 4.22	285 ± 8.89	290 ± 7.74	293 ± 8.87	293 ± 4.97	1.36	0.235	0.862	0.135
PLT (10^9^/L)	452 ± 112	549 ± 53.3	535 ± 130	559 ± 134	572 ± 106	19.33	0.315	0.075	0.427
PCT (%)	0.43 ± 0.13	0.52 ± 0.03	0.50 ± 0.13	0.54 ± 0.14	0.56 ± 0.08	0.02	0.299	0.060	0.728
Day42									
WBC (10^9^/L)	17.3 ± 3.39	19.6 ± 5.30	16.4 ± 4.10	21.3 ± 6.36	23.1 ± 10.2	4.17	0.361	0.258	0.353
Neut (10^9^/L)	1.54 ± 0.36	1.94 ± 0.80	1.17 ± 0.73	1.12 ± 0.58	1.67 ± 0.31	0.19	0.437	0.578	0.345
Lymph (10^9^/L)	10.4 ± 2.72	17.9 ± 3.86	11.6 ± 4.38	16.3 ± 5.80	21.1 ± 14.40	1.87	0.318	0.124	0.699
Mono (10^9^/L)	3.20 ± 1.54	5.49 ± 2.32	2.82 ± 2.51	2.89 ± 2.51	4.50 ± 1.00	0.58	0.492	0.713	0.454
Eos (10^9^/L)	0.31 ± 0.23 ^ab^	0.51 ± 0.08 ^a^	0.21 ± 0.17 ^b^	0.21 ± 0.09 ^b^	0.32 ± 0.18 ^ab^	0.04	0.040	0.245	0.666
Bas (10^9^/L)	0.03 ± 0.01	0.02 ± 0.10	0.03 ± 0.03	0.02 ± 0.02	0.03 ± 0.03	0.04	0.706	0.524	0.354
RBC (10^9^/L)	7.05 ± 0.70	6.92 ± 0.23	6.86 ± 0.69	6.84 ± 0.61	6.77 ± 0.25	0.10	0.917	0.361	0.859
HGB (g/L)	120 ± 12.2	120 ± 6.7	118 ± 5.7	117 ± 7.41	115 ± 4.47	0.09	0.759	0.207	0.781
HCT (%)	41.3 ± 4.35	42.1 ± 2.63	40.6 ± 2.46	40.7 ± 3.84	39.3 ± 1.60	0.56	0.603	0.176	0.573
MCV (fL)	58.6 ± 2.05	60.9 ± 2.43	59.3 ± 3.40	59.5 ± 3.55	58.0 ± 0.73	0.48	0.408	0.430	0.167
RDW-SD (fL)	38.9 ± 2.55	40.5 ± 3.24	41.7 ± 5.85	42.1 ± 2.67	39.1 ± 2.49	1.02	0.434	0.668	0.083
RDW-CV (%)	18.1 ± 1.20	18.2 ± 1.54	19.1 ± 1.65	19.4 ± 2.31	18.4 ± 1.31	0.53	0.550	0.377	0.291
MCH (pg)	17.1 ± 0.58	17.4 ± 0.61	18.9 ± 3.67	17.3 ± 1.38	17.0 ± 0.41	0.17	0.352	0.915	0.130
MCHC (g/L)	291 ± 3.78	285 ± 9.03	291 ± 8.04	290 ± 13.5	294 ± 4.42	1.53	0.515	0.371	0.378
PLT (10^9^/L)	468 ± 109	481 ± 107	478 ± 75.7	362 ± 81.4	433 ± 45.1	16.93	0.135	0.746	0.973
PCT (%)	0.44 ± 0.09	0.47 ± 0.09	0.44 ± 0.08	0.36 ± 0.07	0.41 ± 0.34	0.01	0.218	0.120	0.846

WBC, white blood cell count; Neut, neutrophils; Lymph, lymphocytes; Mono, monocytes; Eos, eosinophils; Bas, basophils; RBC, red blood cell count; HGB, hemoglobin; HCT, hematocrit; MCV, mean corpuscular volume; RDW-SD, standard deviation in red cell distribution width; RDW-CV, coefficient variation of red blood cell volume distribution width; MCH, mean corpuscular hemoglobin; MCHC, mean corpuscular hemoglobin concentration; PLT, platelet; PCT, thrombocytocrit; and CON, basal diet fed. Data are listed as means with SEM (*n* = 6). ^a,b^ mean values with unlike superscript letters were significantly different (*p* < 0.05).

**Table 5:** Effects of CM on serum biochemical parameters in weaned pigs.

Items		CM (mg/kg)				SEM	*p* Value		
	CON	100	1000	2000	4000		ANOVA	Linear	Quadratic
Day1									
TP (g/L)	43.0 ± 4.06	43.2 ± 1.58	42.7 ± 2.17	44.3 ± 2.24	43.2 ± 1.58	0.44	0.837	0.650	0.908
ALB (g/L)	22.2 ± 2.42	21.9 ± 0.81	22.3 ± 1.22	23.6 ± 1.16	21.4 ± 1.58	0.29	0.188	0.970	0.210
GLO (g/L)	20.9 ± 1.72	21.3 ± 1.69	20.4 ± 2.18	20.8 ± 1.42	21.8 ± 1.18	0.30	0.629	0.520	0.322
ALP (U/L)	162 ± 29.0	151 ± 44.1	164 ± 32.0	146 ± 29.3	159 ± 49.5	6.82	0.925	0.839	0.799
ALT (U/L)	58.6 ± 8.28	52.1 ± 6.03	55.7 ± 9.83	54.4 ± 14.7	51.7 ± 5.53	1.67	0.721	0.358	0.857
AST (U/L)	34.1 ± 10.4	36.2 ± 12.5	37.2 ± 5.84	38.8 ± 10.4	41.3 ± 12.1	1.84	0.808	0.029	0.404
CRE (umol/L)	60.7 ± 13.1	75.9 ± 9.04	65.2 ± 7.60	66.2 ± 8.94	58.4 ± 16.6	2.26	0.12	0.346	0.065
GLU (mmol/L)	4.53 ± 0.46	4.35 ± 0.63	4.48 ± 0.48	5.12 ± 0.85	4.23 ± 0.33	0.11	0.109	0.815	0.316
TBIL (umol/L)	0.17 ± 0.19	0.69 ± 0.93	0.34 ± 0.27	0.34 ± 0.27	0.53 ± 0.71	0.144	0.451	0.475	0.001
UREA (mmol/L)	2.76 ± 1.49	2.94 ± 0.99	2.51 ± 1.17	3.13 ± 0.75	2.60 ± 0.68	0.18	0.848	0.930	0.530
Day42									
TP (g/L)	49.5 ± 2.55	49.3 ± 3.60	51.0 ± 2.74	50.6 ± 1.96	48.8 ± 2.02	0.47	0.583	0.992	0.207
ALB (g/L)	29.3 ± 1.75	28.6 ± 4.74	29.5 ± 2.28	29.6 ± 2.19	28.4 ± 3.01	0.51	0.937	0.838	0.686
GLO (g/L)	20.2 ± 2.07	20.6 ± 1.56	21.4 ± 1.75	21.0 ± 2.45	20.4 ± 1.87	0.34	0.816	0.746	0.274
ALP (U/L)	143 ± 16.5	119 ± 7.89	143 ± 22.8	145 ± 21.7	148 ± 8.22	3.71	0.085	0.681	0.643
ALT (U/L)	56.8 ± 5.34	52.2 ± 9.77	52.5 ± 6.54	55.1 ± 13.5	56.7 ± 7.11	1.56	0.833	0.817	0.295
AST (U/L)	30.1 ± 10.4	36.2 ± 12.4	37.2 ± 5.84	38.8 ± 10.4	41.3 ± 12.1	1.84	0.808	0.223	0.929
CRE (umol/L)	77.4 ± 18.5	80.7 ± 15.6	86.5 ± 7.89	84.6 ± 13.4	69. ± 113.5	2.67	0.258	0.490	0.047
GLU (mmol/L)	5.22 ± 1.15	5.72 ± 1.43	4.23 ± 0.65	4.84 ± 1.03	4.41 ± 1.13	0.21	0.163	0.092	0.047
TBIL (umol/L)	0.17 ± 0.19	0.69 ± 0.93	0.93 ± 1.14	0.34 ± 0.27	0.53 ± 0.71	0.14	0.451	0.698	0.205
UREA (mmol/L)	2.79 ± 0.77	2.32 ± 0.54	2.67 ± 0.63	3.00 ± 0.63	2.54 ± 0.90	0.13	0.543	0.850	0.991

TP, total protein; ALB, albumin; GLO, globulin; ALP, alkaline phosphatase; ALT, alanine aminotransferase; AST, aspartate aminotransferase; CRE, creatinine; GLU, blood glucose; TBIL, bilirubin total; and CON, basal diet fed. Data are listed as means with SEM (*n* = 6).

**Table 6:** Effects of CM on serum antioxidant indices in weaned pigs.

Items		CM (mg/kg)				SEM	*p* Value		
	CON	100	1000	2000	4000		ANOVA	Linear	Quadratic
Day1									
MDA (nmol/mg)	3.48 ± 0.89	3.45 ± 0.42	3.67 ± 0.73	4.05 ± 0.69	3.63 ± 0.43	0.12	0.532	0.294	0.541
GSH-Px (μg/mg)	375 ± 33.0	384 ± 70.7	320 ± 107	393 ± 62.9	340 ± 65.7	13.21	0.364	0.503	0.902
CAT (U/mg)	2.20 ± 1.28	2.90 ± 0.98	3.52 ± 1.82	3.38 ± 2.41	3.43 ± 1.31	0.29	0.610	0.175	0.415
T-AOC (U/mg)	5.50 ± 2.05	5.01 ± 1.92	4.22 ± 0.81	4.59 ± 0.88	3.91 ± 1.01	0.23	0.495	0.104	0.762
SOD (U/mg)	168 ± 6.20	170 ± 7.26	168 ± 10.47	165 ± 10.79	167 ± 4.70	1.43	0.877	0.578	0.993
Day42									
MDA (nmol/mg)	7.83 ± 2.76 ^a^	4.84 ± 1.15 ^b^	8.11 ± 0.47 ^a^	9.13 ± 2.07 ^a^	7.23 ± 1.68 ^a^	0.41	0.011	0.209	0.982
GSH-Px (μg/mg)	415 ± 34.3	417 ± 85.7	372 ± 60.5	348 ± 39.6	384 ± 91.0	12.28	0.337	0.153	0.338
CAT (U/mg)	5.56 ± 2.75 ^bc^	9.95 ± 1.66 ^a^	7.38 ± 2.89 ^ab^	3.50 ± 1.73 ^c^	3.58 ± 2.75 ^c^	0.65	0.001	0.005	0.021
T-AOC (U/mg)	3.71 ± 0.88 ^b^	5.35 ± 1.53 ^a^	3.86 ± 0.81 ^b^	3.81 ± 0.88 ^b^	3.08 ± 1.01 ^b^	0.23	0.015	0.050	0.050
SOD (U/mg)	171 ± 11.2	171 ± 6.9	172 ± 8.68	172 ± 9.43	170 ± 5.49	1.46	0.983	0.992	0.592

CAT, catalase; T-AOC, total antioxidant capacity; SOD, superoxide dismutase; GSH-Px, glutathione peroxidase; MDA: malondialdehyde; and CON, basal diet fed. Data are listed as means with SEM (*n* = 6). ^a,b,c^ mean values with unlike superscript letters were significantly different (*p* < 0.05).

**Table 7:** Effect of CM on relative weights of the organ in pigs.

Items		CM (mg/kg)				SEM	*p* Value		
	CON	100	1000	2000	4000		ANOVA	Linear	Quadratic
Heart	0.51 ± 0.04	0.50 ± 0.05	0.49 ± 0.04	0.51 ± 0.02	0.52 ± 0.05	0.06	0.550	0.550	0.499
Liver	2.77 ± 0.21	2.67 ± 0.13	2.89 ± 0.21	2.84 ± 0.11	2.91 ± 0.28	0.04	0.282	0.282	0.028
Spleen	0.22 ± 0.03	0.20 ± 0.03	0.20 ± 0.02	0.22 ± 0.03	0.21 ± 0.05	0.01	0.840	0.840	0.867
Lung	1.65 ± 0.43	1.38 ± 0.22	1.28 ± 0.67	1.38 ± 0.22	1.52 ± 0.16	0.07	0.518	0.518	0.591
Kidney	0.26 ± 0.03	0.24 ± 0.04	0.25 ± 0.01	0.24 ± 0.02	0.27 ± 0.03	0.01	0.153	0.251	0.050
Pancreas	0.11 ± 0.03	0.10 ± 0.03	0.10 ± 0.02	0.11 ± 0.02	0.12 ± 0.21	0.04	0.712	0.712	0.306

CON, basal diet fed. Data are listed as means with SEM (*n* = 6).

**Table 8:** Composition and nutrient level of experimental diets (as fed basis).

Ingredients, %	Days 1–21	Days 22–42
Corn	35.36	38.41
Extruded corn	23.94	30.52
Soybean meal	6.42	12.00
Extruded soybean	9.00	10.25
Fish meal	4.20	5.00
Whey powder	8.00	0.00
Soybean protein concentrate	8.40	0.00
Soybean oil	1.70	1.34
Sucrose	0.47	0
Limestone	0.53	0.60
Dicalcium phosphate	0.60	0.40
NaCl	0.30	0.30
L-Lysine HCl, 78%	0.30	0.35
DL-Methionine	0.08	0.08
L-Threonine, 98.5%	0.03	0.05
Tryptophan, 98%	0.02	0.05
Chloride choline	0.10	0.10
Vitamin premix ^a^	0.05	0.05
Mineral premix ^b^	0.30	0.30
Total	100.00	100.00
Nutrient concentration ^c^		
DE (MJ/kg)	3.53	3.49
CP, %	19.52	18.10
Ca, %	0.80	0.72
Total P, %	0.62	0.55
Available P, %	0.43	0.36
D-Lysine, %	1.37	1.24
D-Methionine, %	0.41	0.39
D-Threonine, %	0.83	0.74
D-Tryptophan, %	0.24	0.24

^a^ The vitamin premix provided following per kilogram of diet: VitaminA, 15,000 IU; VitaminD3, 3000 IU; VitaminE, 40 mg; VitaminK3, 5.0 mg; VitaminB2, 12.5 mg; and VitaminB12, 0.3 mg. ^b^ The mineral premix provided following per kilogram of diet: Mn, 2 mg/kg; Cu, 6 mg/kg; I, 0.14 mg/kg; Zn, 100 mg/kg; Fe, 60 mg/kg; and Se, 0.35 mg/kg. ^c^ Calculated nutrient levels.

## Data Availability

The original contributions presented in the study are included in the article, further inquiries can be directed to the corresponding authors.
